# Effect of Elemental Sulfur and Sulfide on the Corrosion Behavior of Cr-Mo Low Alloy Steel for Tubing and Tubular Components in Oil and Gas Industry

**DOI:** 10.3390/ma10040430

**Published:** 2017-04-20

**Authors:** Ladan Khaksar, John Shirokoff

**Affiliations:** 1 Department of Mechanical Engineering, Faculty of Engineering and Applied Science, Memorial University of Newfoundland, St. John’s, NL A1B 3X5, Canada; 2 Department of Process Engineering, Faculty of Engineering and Applied Science, Memorial University of Newfoundland, St. John’s, NL A1B 3X5, Canada; shirokof@mun.ca

**Keywords:** corrosion behavior, elemental sulfur, sulfide, 4130 Cr-Mo alloy, potentiodynamic polarization

## Abstract

The chemical degradation of alloy components in sulfur-containing environments is a major concern in oil and gas production. This paper discusses the effect of elemental sulfur and its simplest anion, sulfide, on the corrosion of Cr-Mo alloy steel at pH 2 and 5 during 10, 20 and 30 h immersion in two different solutions. 4130 Cr-Mo alloy steel is widely used as tubing and tubular components in sour services. According to the previous research in aqueous conditions, contact of solid sulfur with alloy steel can initiate catastrophic corrosion problems. The corrosion behavior was monitored by the potentiodynamic polarization technique during the experiments. Energy dispersive X-ray spectroscopy (EDS) and scanning electron microscopy (SEM) have been applied to characterize the corrosion product layers after each experiment. The results show that under the same experimental conditions, the corrosion resistance of Cr-Mo alloy in the presence of elemental sulfur is significantly lower than its resistance in the presence of sulfide ions.

## 1. Introduction

For more than 40 years, elemental sulfur deposition in pipelines and facilities has become a major concern in the sour oil and gas industry [1]. In conjunction with reservoir souring, the incidence of sulfur corrosion will likely increase. It is known from prior research that the presence of dry elemental sulfur in contact with carbon steel is not considered as a corrosion threat to steel; however, by adding water to the system, the corrosion process may be dramatically accelerated [2].

Elemental sulfur usually appears in an aqueous system due to the oxidation of sulfide species where the possible reaction for the formation of elemental sulfur (S_8_) may involve high oxidation state metals or oxygen [3]:

8 H_2_S (aq) + 16 M^n+^ (aq) → S_8_ (s) + 16 H^+^ (aq) + 16 M^(n−1)+^ (aq) (1)

8 H_2_S (aq) + 4 O_2_ (g) → S_8_ (s) + 8 H_2_O (l(2)) (2)

MacDonald et al. hypothesized that an electrochemical reaction between iron and polysulfide could be the driving force for a corrosion process where elemental sulfur is present [4]:

(x − 1)Fe (s) + S_y−1_ S^2−^ (aq) + 2 H^+^ (aq) → (x − 1) FeS (s) + H_2_S (g) + S_y−x_ (s) (3)

In recent years, Fang et al. investigated the corrosion behavior of carbon steel at different temperatures with molten sulfur on the steel surface [5,6]. These investigations comprehensively studied the sulfur hydrolysis and direct sulfur/iron reaction, with either an electrically insulating or conductive barrier placed between the sulfur droplet and the metal surface.

The investigation of Fang et al. proved that the electrical connection and physical proximity between sulfur and steel are critical characteristics for elemental sulfur corrosion of mild steel. They also identified that an electrochemical reaction is the likely mechanism of elemental sulfur corrosion of mild steel. However, there are few electrochemical investigations on the corrosion behavior of an alloy steel such as 4130 in the presence of elemental sulfur. In this paper, the effect of elemental sulfur and its anion on corrosion mechanism and the behavior of the Cr-Mo low alloy steel were investigated at varying pH levels and immersion time through corrosion simulation tests and electrochemical measurements. 

## 2. Experimental Procedure

### 2.1. Material and Sample Preparation

According to National Association of Corrosion Engineering (NACE) MR0175/ISO 15156, the most common steel alloy for tubular and tubular components in sour service is Unified Number System (UNS) G41XX0, formerly American Iron and Steel Institute (AISI) 41XX [7]. 4130 steel is among the most common low alloys used in the oil and gas industry. This steel typically consists of 0.80–1.1 Cr, 0.15–0.25 Mo, 0.28–0.33 C, 0.40–0.60 Mn, 0.035 P, 0.040 S, 0.15–0.35 Si and balance Fe. The working electrode was machined from the parent material into cylinders having dimensions of approximately 9 mm in length and 9 mm in diameter. Prior to the experiments, all specimens were polished with Coated Abrasive Manufacturers Institute (CAMI) grit designations 320, 600 and 1000, corresponding to average particle diameters 36.0, 16.0 and 10.3 microns and finally 6-micron grit silicon carbide paper, then cleansed with deionized water until a homogeneous surface was observed. Following this, the specimens were quickly dried using cold air to avoid oxidation.

After preparing the samples, they were transferred into a multi-port glass cell, which was filled with 3.5% sodium chloride solution. The pH was adjusted by adding deoxygenated hydrochloric acid or sodium hydroxide. Prior to the start of each electrochemical test, the sample was immersed in the solution for 55 min in accordance with ASTM G5-82 [8].

### 2.2. Direct Sulfur/Iron and Sulfide/Iron Reactions Preparation

Two series of experiments have been performed to investigate the effects of elemental sulfur (S8) and its simplest anion, sulfide (S^2−^), on the corrosion behavior of Cr-Mo low alloy steel at varying pH and immersion times. In the first series of experiments, all of the tests were carried out in a multi-port glass cell, which was filled with solved thioacetamide (2 M) in 420 mL de-ionized water. According to the literature and the authors’ previous studies [9,10], the addition of thioacetamide into the water would produce free sulfide ions through the bulk solution. Decomposition of thioacetamide is an irreversible reaction that has been considered as the sulfur source, generating S^2−^ by a hydrolytic method [11,12,13,14]. The following equation shows the presence of dissolved free sulfides in di-ionized water, which are super active to react with samples. Table 1 describes the experimental conditions of the first series of experiments.

In the second series, a similar method to the method of Fang et al. with maximum uniform coverage of adherent sulfur to the coupon surface was employed for all of the tests [1,5,15]. In this series of experiments, samples’ surfaces were covered with sublimed elemental sulfur 99.9999% (ACROS) deposited onto polished samples. Table 2 describes the experimental conditions of the second series of experiments.

CH_3_CSNH_2_ + H_2_O ↔ S^2−^ + CH_3_CONH_2_ + 2H^+^ (4)

### 2.3. Electrochemical Measurements

Electrochemical corrosion experiments, and in particular the potentiodynamic polarization scan, can provide considerable information on the corrosion rate, pitting susceptibility and passivity, as well as the cathodic behavior of an electrochemical system [16]. During this study, experiments were conducted in a multi-port glass cell with a three-electrode setup at atmospheric pressure based on the ASTM G5-82 standard for potentiodynamic anodic polarization measurements [8].

A graphite rod was used as the counter electrode (CE) and saturated silver/silver chloride (Ag/AgCl) was used as the reference electrode (RE). Furthermore, as was mentioned in the material and sample preparation, 4130 low alloy steel was used as the working electrode (WE).

An Ivium Compactstat Potentiostat monitoring system was used to perform electrochemical corrosion measurements. The potentiodynamic polarization technique was applied to investigate the corrosion behavior. The applied scan rate for this measurements was 0.125 mV/s. 

### 2.4. Surface Morphology Observation and Corrosion Product Layers Analysis

Upon completion of corrosion testing, morphological characterization of the surface was conducted using an FEI Quanta 400 scanning electronic microscope (SEM) with Bruker energy dispersive X-ray (EDS) spectroscopy. The SEM was operating at 15 kV, with a working distance of 15 mm and beam current of 13 nA.

## 3. Results and Discussion 

### 3.1. First Series of Experiments; Effect of Sulfide (S^2−^) on the Corrosion Mechanism of Cr-Mo Low Alloy Steel 

As was mentioned in the experimental procedure, in order to investigate the effect of sulfide (S^2−^) on the corrosion behavior of 4130 alloy, the samples were immersed into the solution containing solved thioacetamide (2 M) in 420 mL de-ionized water for 10, 20 and 30 h at 80 °C, pH 2 and 5.

#### 3.1.1. Corrosion Behavior of Cr-Mo Low Alloy Steel

**Figure 1:**
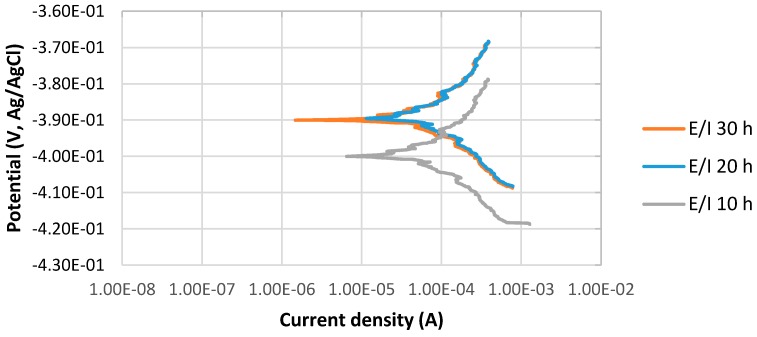
The potentiodynamic curves of 4130 Cr-Mo alloy steel in thioacetamide solution at different immersion times: 10, 20 and 30 h at 80 °C, pH 2.

The potentiodynamic curves of 4130 Cr-Mo low alloy steel in thioacetamide solution at different immersion times, 10, 20 and 30 h at 80 °C, pH 2, are illustrated in Figure 1. The scan rate was 0.125 mV/s.

Figure 1 indicates the stable behavior of anodic curves with increasing the immersion time from 10 to 30 h at 80 °C, pH 2. It illustrates that the corrosion potential, E_corr_, at pH 2 for 20 and 30 h immersion time is almost the same and more positive than that of 10 h immersion in the solution; however, the values of difference are not significant. It can be also observed that the current density of 10 h immersion is higher than those of 20 and 30 h immersion.

**Figure 2:**
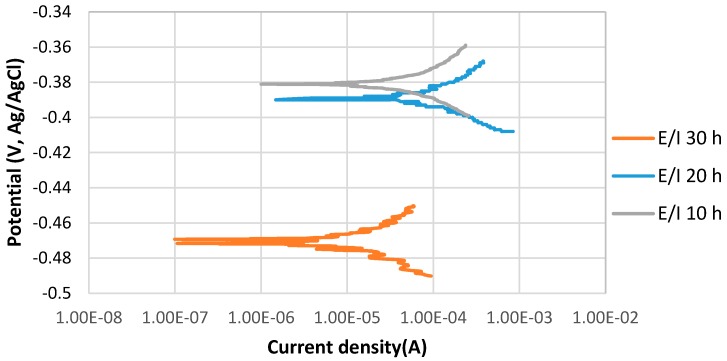
The potentiodynamic curves of 4130 Cr-Mo alloy steel in thioacetamide solution at different immersion times: 10, 20 and 30 h at 80 °C, pH 5.

Figure 2 shows the stable behavior of anodic curves with increasing the immersion time from 10 to 30 h at 80 °C, pH 5. The potentiodynamic polarization curves indicate that E_corr_ of 10 h immersion was relatively more positive than that of 30 h immersion, which was more positive than that of 20 h. The current density of 10 h immersion is slightly lower than that of the 30 h immersion time, which is significantly higher than the current density of 20 h immersion in the solution. 

During a corrosion process, the rate of the reactions is determined by the corrosion mechanism. The growth of a corrosion product layer limits the rate of further corrosion by acting as a diffusion barrier for the species involved in the process [17,18]. After 20 h immersion at pH 5, the formation of a protective corrosion product layer prevented the further corrosion of the sample surface; however, after 30 h immersion, the corrosion current density significantly increased, which may be related to the breaking down of the protective corrosion product layer on the alloy surface. The values of anodic ($\beta_{a}$) and cathodic ($\beta_{c}$) Tafel slopes of the samples of each experiment were obtained by potentiostat as illustrated in Table 3. 

#### 3.1.2. Corrosion Rate of Cr-Mo Low Alloy Steel

The corrosion current (*i_corr_*) was calculated using the following equations [19]:

(5) $$i_{corr}=\frac{B}{R_{P}}$$

where, $i_{corr}$ is the corrosion current density in A·$m^{-2}$; $R_{P}$ is the polarization resistance in Ω·$m^{2}$ and *B* is the proportionality constant in mV·decade^–1^:

(6) $$B=\;\frac{\beta_{a}\;\beta_{c}}{2.3\;\left(\beta_{a}+\;\beta_{c}\right)\;}$$

which can be calculated by the given values of anodic ($\beta_{a}$) and cathodic ($\beta_{c}$) Tafel slopes of the samples of each experiment.

Finally, the corrosion rate (CR) was calculated using the following equation:

(7) $$\mathrm{CR}=\frac{i_{corr}\;w}{\rho \;F}$$

where, *w* is the equivalent weight of 4130 alloy; *F* is the Faraday constant, and *ρ* is the density of 4130 alloy.

Table 4 indicates the corrosion rate of each experiment.

As can be observed in Table 4, in thioacetamide solution, the corrosion rate of Cr-Mo alloy at pH 2 is greater than that of pH 5, which is usually related to the formation of a corrosion protective layer at higher pH. At pH 2, iron is dissolved, and iron sulfide is not significantly precipitated on the surface of the alloy due to the high solubility of iron sulfide phases at pH values less than 2 [20,21,22]. In this case, sulfide exhibits only the accelerating effect on the dissolution of iron. At pH 5, the inhibitive effect of sulfide is seen due to the formation of iron sulfide protective film on the alloy surface [16].

Table 4 shows that the corrosion rate has a maximum of 0.368 mm/year after 10 h immersion at pH 2, which slightly decreases to 0.318 mm/year after 30 h immersion. The corrosion rates of pH 5 indicate a small decrease and a large increase during 20 and after 30 h immersion, respectively, due to the formation and breakdown of the corrosion product layer. These results are consistent with data obtained from the potentiodynamic polarization technique.

### 3.2. Second Series of Experiments: the Effect of Elemental Sulfur (S_8_) on the Corrosion Mechanism of Cr-Mo Low Alloy Steel 

As was mentioned in the experimental procedure, in order to investigate the effect of elemental sulfur (S_8_) on the corrosion behavior of 4130 Cr-Mo alloy, the surfaces of the samples were covered by melted elemental sulfur 99.999% (ACROS) and then immersed in a glass cell, which was filled with the 3.5% sodium chloride solution.

#### 3.2.1. Corrosion Behavior of Cr-Mo Low Alloy Steel 

**Figure 3:**
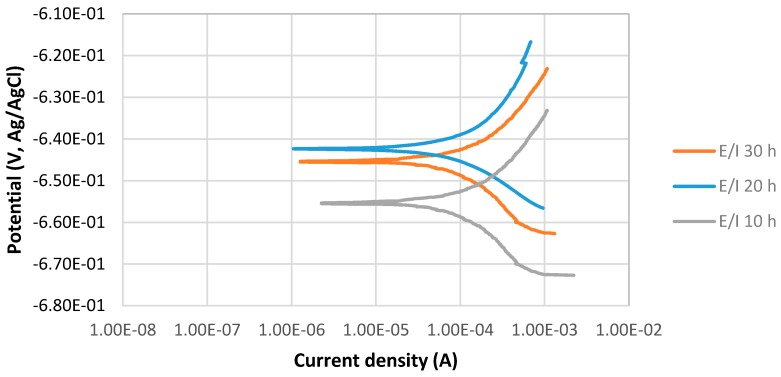
The potentiodynamic curves of 4130 Cr-Mo alloy steel covered with elemental sulfur in 3.5% sodium chloride solution at different immersion times: 10, 20 and 30 h at 80 °C, pH 2.

The potentiodynamic curves of 4130 Cr-Mo low alloy steel covered with elemental sulfur in the 3.5% sodium chloride solution at different immersion times, 10, 20 and 30 h at 80 °C, pH 2, are illustrated in Figure 3. The scan rate was 0.125 mV/s. Figure 3 presents that E_corr_ at pH 2 for the 20 h immersion time is more positive than that of 30 h immersion in the solution; however, the difference is not significant. It can be observed that the current density of 30 h immersion is higher than that of 20 h immersion.

Figure 3 also shows that E_corr_ at pH 2 for 10 h immersion time is the most negative one. It can be observed that current density of 10 h immersion is higher than that at 20 and 30 h immersion.

**Figure 4:**
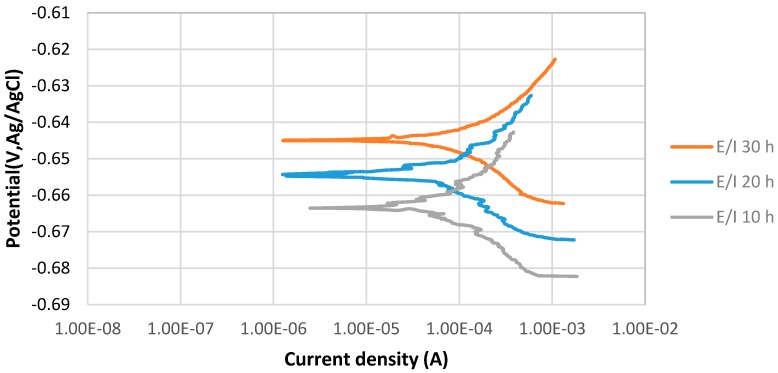
The potentiodynamic curves of 4130 Cr-Mo alloy steel covered with elemental sulfur in 3.5% sodium chloride solution at different immersion times: 10, 20 and 30 hat pH 5.

Figure 4 shows the stable behavior of anodic curves of samples covered with elemental sulfur with increasing the immersion time from 10 to 30 h at 80 °C and pH 5. The potentiodynamic polarization curves indicate that E_corr_ of 30 h immersion is more positive than that of 20 h immersion in the 3.5% sodium chloride solution. It can be observed that current density of 20 h immersion is higher than that of 30 h immersion. The values of anodic ($\beta_{a}$) and cathodic ($\beta_{c}$) Tafel slopes of the samples of each experiments were determined as illustrated in Table 5.

#### 3.2.2. Corrosion Rate of Cr-Mo Low Alloy Steel

The corrosion rates of the second series of the experiments were calculated with the same method as the first series. Table 6 indicates the corrosion rate of each experiment.

As Table 6 indicated, generally the corrosion rates of Cr-Mo alloy in the presence of elemental sulfur are greater than those in the presence of sulfide ions. Furthermore, it can be observed that at pH 2, the rates of corrosion are higher than those of pH 5, which is due to the formation of protective corrosion product layers on the alloy surface at pH greater than 2. The corrosion rate after 10 h immersion at pH 2 has a maximum of 0.615 mm/year, which slightly decreased to 0.595 mm/year after 30 h immersion. The corrosion rates of pH 5 gradually decreased by increasing the immersion time due to the formation of protective corrosion product layer. These results are consistent with data obtained from the potentiodynamic polarization technique.

### 3.3. Analysis of Corrosion Product Layers on the Surface of the Alloy

**Figure 5:**
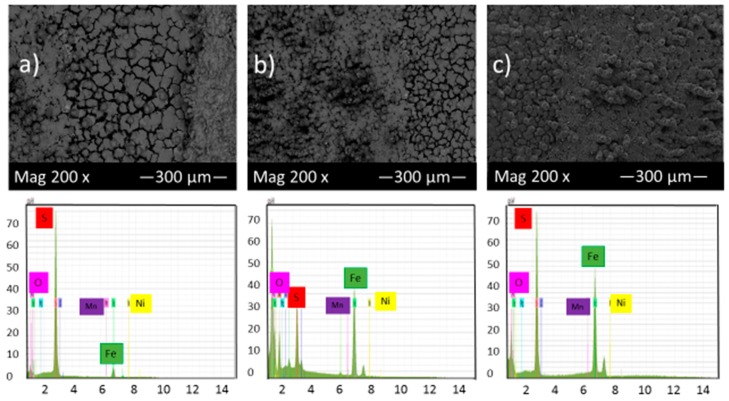
SEM micrograph and EDS of the corrosion product layers that form on the surface of each sample at pH 2 under (**a**) 10, (**b**) 20 and (**c**) 30 h immersion time in thioacetamide solution.

Figure 5 shows the SEM micrograph of the corrosion product layers that form on the surface of each sample at pH 2 under 10, 20 and 30 h immersion time in thioacetamide solution. Figure 5 shows that by increasing the immersion time, a thin corrosion product layer gradually covered the alloy surface and protected it from further corrosion. EDS results indicate that this corrosion product layer contains iron and sulfur and so, likely, compounds of iron sulfide.

The EDS spectrum of Figure 5a illustrates that once the corrosion product layer formed on the sample surface, in some areas, the elemental sulfur was observed as the predominant constituent with a high ratio compared to elemental iron. The same features have been reported by F. Alabbas et al. [23].

**Figure 6:**
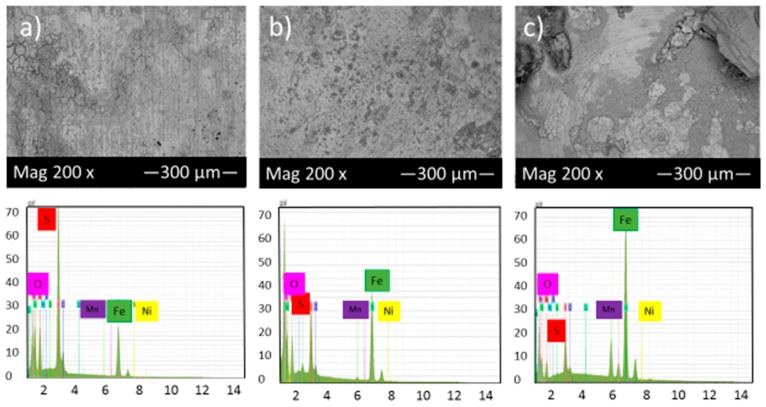
SEM micrograph and EDS of the corrosion product layers that form on the surface of each sample at pH 5 under (**a**) 10, (**b**) 20 and (**c**) 30 h immersion time in thioacetamide solution.

Figure 6 shows the SEM micrograph of the corrosion product layers that form on the surface of each sample at pH 5 under 10, 20 and 30 h immersion time in thioacetamide solution.

Figure 6a,b shows that generally, a much thicker film was deposited on the alloy surface at pH 5 after 10 and 20 h immersion in thioacetamide solution. The composition of this film was shown by EDS to consist of iron and sulfur. After 30 h immersion in the solution, the corrosion product layer was broken and exposed the sample surface to the corrosive solution. 

Comparison of Figure 5 and Figure 6 and also the cross-section of the corrosion product layers show that at pH 2, a very thin and open structure layer formed, which could not display a protective role against corrosion. However, at pH 5, the corrosion product layer was more dense, adherent and protective due to a higher volume of precipitated products on the sample surface.

Higher pH would generally decrease the solubility of the corrosion products layer and consequently result in an increase of precipitation rate, faster formation of protective layers and the reduction of corrosion rates [21]. 

**Figure 7:**
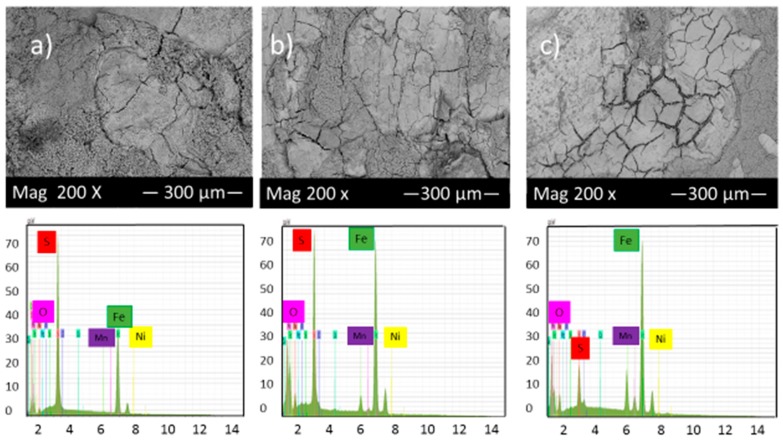
SEM micrograph and EDS of the corrosion product layers that form on the surface of each sample covered with elemental sulfur at pH 2 under (**a**) 10, (**b**) 20 and (**c**) 30 h immersion time.

Figure 7 shows the SEM micrograph of the corrosion product layers that formed on the surface of each sample covered with elemental sulfur at pH 2 under 10, 20 and 30 h immersion time.

Figure 7 illustrated that the highest percentage of cracks and pits can be observed in these experimental conditions; however, the corrosion product layers’ formation on the alloy surface gradually increased with time, which slightly reduced the corrosion rate. The EDS analysis shows the presence of different values of iron and sulfur, which also indicates the presence of various compounds of iron sulfide on the surface of samples.

**Figure 8:**
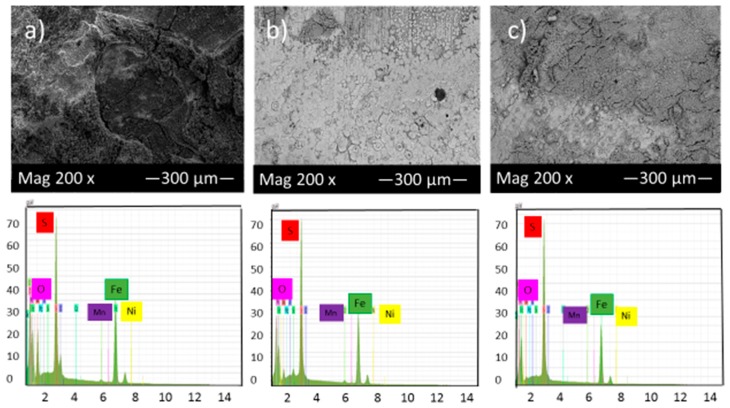
SEM micrograph of the corrosion product layers that form on the surface of each sample covered with elemental sulfur at pH 5 under (**a**) 10, (**b**) 20 and (**c**) 30 h immersion time.

Figure 8 shows the SEM micrograph of the corrosion product layers that form on the surface of each sample covered with elemental sulfur at pH 5 under 10, 20 and 30 h immersion time.

Comparison of Figure 7 and Figure 8 shows that by increasing pH from 2 to 5, the corrosion product layer became more even and continuous, which is consistent with data from corrosion rate and potentiodynamic polarization tests. At pH 5 and after 30 h immersion in the solution, the corrosion product layers became finer and compact, indicative of good protection for the alloy compared to those of 10 and 20 h immersion. The formation of this condensed corrosion product layer slightly prevents further corrosion and consequently decreases the corrosion rate with time. 

#### General Comparison of the Corrosion Product Layers in Two Series of Experiments

**Figure 9:**
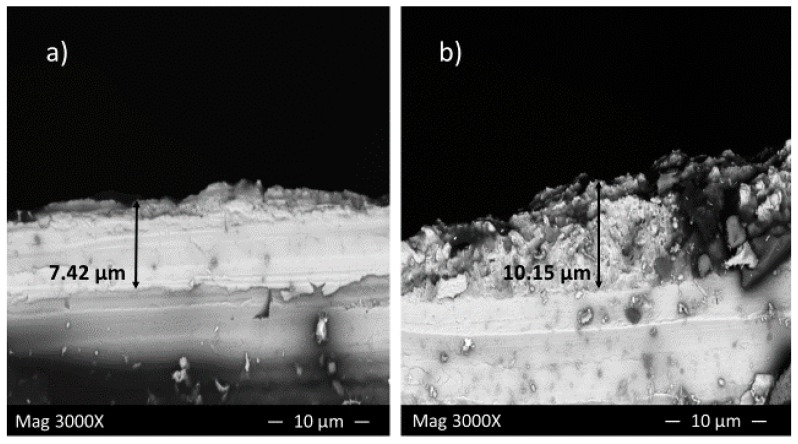
Cross-section of corrosion product layer of the (**a**) first and (**b**) second series of experiments at pH 5 after 10 h immersion time.

Figure 9a,b shows the cross-section of corrosion product layers of the first and second series of experiments at pH 5 after 10 h immersion time, respectively. 

In the presence of sulfide ions, Figure 9a, a thin, dense and adherent layer covered the sample surface with a thickness of approximately 7 µm, which provided a barrier against further corrosion; however, in the presence of elemental sulfur, Figure 9b, the top surface layer indicates a flaky structure. The thickness of this layer is about 10.15 µm, which still cannot provide enough protection due to the structure being too porous and detached from the sample surface. 

The results of cross-sectional analysis verified the results from the corrosion rate calculation and potentiodynamic measurements.

**Figure 10:**
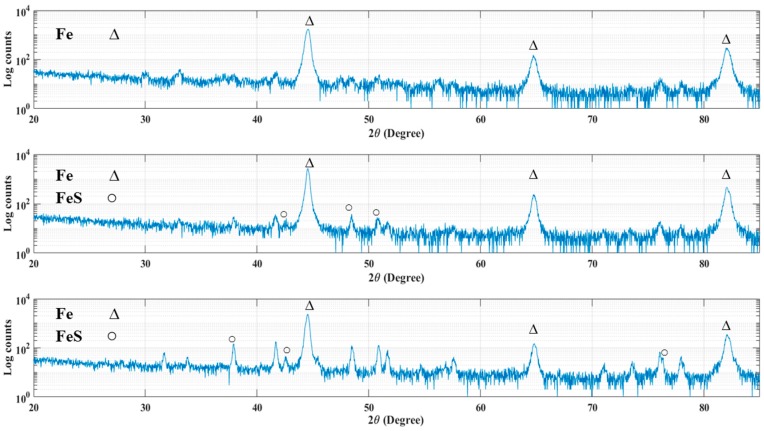
XRD pattern for the samples in (**a**) the second series of experiment at pH 5 after 30 h immersion, (**b**) the first series of experiments at pH 5 after 10 h immersion and (**c**) the second series of experiment at pH 5 after 10 h immersion.

The XRD patterns of 4130 Cr-Mo alloy steel exposed to sulfide ions and elemental sulfur are displayed in Figure 10. As has been mentioned in the cross-sectional analysis, the corrosion product layer thickness is extremely low for most of the samples, which made them undetectable with XRD measurements. Figure 10a indicates the XRD pattern for the sample covered with elemental sulfur at pH 5 after 30 h immersion. As can be seen, iron is the only element that was detected on the sample surface.

The XRD patterns in Figure 10b,c confirmed the formation of iron sulfide compounds on the surface of the samples at pH 5 after 10 h immersion time in the first and second series of experiments respectively, where the corrosion product layers were thick enough to be detected by X-ray spectra.

## 4. Conclusions

- Corrosion resistance of Cr-Mo alloy in the presence of elemental sulfur is significantly lower than its resistance in the presence of sulfide ions with the same experimental conditions.
- Increasing the pH significantly decreases the corrosion rate of Cr-Mo alloy steel in the presence of elemental sulfur, which is due to the formation of evener and compact corrosion product layers on the alloy surface.
- The effect of immersion time on the corrosion behavior of the alloy is more complicated than the effect of pH. Results suggest that a number of factors such as microstructure, composition and the stability of corrosion product layers and immersion time can increase or decrease the corrosion rate.
- How stable the corrosion product layers are from elemental sulfur corrosion in various aggressive environments needs to be further investigated.

## Figures and Tables

**Table 1:** Experimental condition of the first series.

Condition No.	T (°C)	pH	Immersion Time (h)
1	80	2	10
2	80	2	20
3	80	2	30
4	80	5	10
5	80	5	20
6	80	5	30

**Table 2:** Experimental condition of the second series.

Condition No.	T (°C)	PH	Immersion Time (h)
7	80	2	10
8	80	2	20
9	80	2	30
10	80	5	10
11	80	5	20
12	80	5	30

**Table 3:** The values of anodic ($\beta_{a}$) and cathodic ($\beta_{c}$) Tafel slopes of first series.

Experiment	$\beta_{a}$ (mV·Decade^−1^)	$\beta_{c}$ (mV·Decade^−1^)
1	0.022	0.019
2	0.029	0.020
3	0.020	0.019
4	0.034	0.023
5	0.021	0.018
6	0.028	0.020

**Table 4:** The corrosion rate of the first series.

Experiment	1	2	6	4	5	6
pH	2	2	2	5	5	5
Corrosion Rate (CR) (mm/year)	0.368	0.325	0.318	0.066	0.044	0.224

**Table 5:** The values of anodic ($\beta_{a}$) and cathodic ($\beta_{C}$) Tafel slopes of second series.

Experiment	$\beta_{a}$ (mV·Decade^−1^)	$\beta_{C}$ (mV·Decade^−1^)
7	0.032	0.015
8	0.030	0.013
9	0.023	0.020
10	0.022	0.021
11	0.022	0.019
12	0.020	0.019

**Table 6:** The corrosion rates of second series.

Experiment	7	8	9	10	11	12
pH	2	2	2	5	5	5
Corrosion Rate (CR) (mm/year)	0.615	0.605	0.595	0.381	0.367	0.318
